# *Bordetella* Adenylate Cyclase Toxin Elicits Airway Mucin Secretion through Activation of the cAMP Response Element Binding Protein

**DOI:** 10.3390/ijms22169064

**Published:** 2021-08-23

**Authors:** Anna Malandra, Waheed Ur Rahman, Nela Klimova, Gaia Streparola, Jana Holubova, Adriana Osickova, Simone Bariselli, Peter Sebo, Radim Osicka

**Affiliations:** 1 Institute of Microbiology of the Czech Academy of Sciences, Videnska 1083, 142 20 Prague 4, Czech Republic; ingamalandra@gmail.com (A.M.); waheed.rahman@biomed.cas.cz (W.U.R.); nelaklim@biomed.cas.cz (N.K.); g.streparola@gmail.com (G.S.); hejnova@biomed.cas.cz (J.H.); osickova@biomed.cas.cz (A.O.); 2 Czech Centre for Phenogenomics, Institute of Molecular Genetics of the Czech Academy of Sciences, Prumyslova 595, 252 50 Vestec, Czech Republic; simone.bariselli@izsler.it

**Keywords:** adenylate cyclase toxin, *Bordetella*, cAMP, CREB, epithelium, mucin, pertussis toxin

## Abstract

The mucus layer protects airway epithelia from damage by noxious agents. Intriguingly, *Bordetella pertussis* bacteria provoke massive mucus production by nasopharyngeal epithelia during the initial coryza-like catarrhal stage of human pertussis and the pathogen transmits in mucus-containing aerosol droplets expelled by sneezing and post-nasal drip-triggered cough. We investigated the role of the cAMP-elevating adenylate cyclase (CyaA) and pertussis (PT) toxins in the upregulation of mucin production in *B. pertussis-*infected airway epithelia. Using human pseudostratified airway epithelial cell layers cultured at air–liquid interface (ALI), we show that purified CyaA and PT toxins (100 ng/mL) can trigger production of the major airway mucins Muc5AC and Muc5B. Upregulation of mucin secretion involved activation of the cAMP response element binding protein (CREB) and was blocked by the 666-15-Calbiochem inhibitor of CREB-mediated gene transcription. Intriguingly, a *B. pertussis* mutant strain secreting only active PT and producing the enzymatically inactive CyaA-AC^–^ toxoid failed to trigger any important mucus production in infected epithelial cell layers in vitro or in vivo in the tracheal epithelia of intranasally infected mice. In contrast, the PT^–^ toxoid-producing *B. pertussis* mutant secreting the active CyaA toxin elicited a comparable mucin production as infection of epithelial cell layers or tracheal epithelia of infected mice by the wild-type *B. pertussis* secreting both PT and CyaA toxins. Hence, the cAMP-elevating activity of *B. pertussis*-secreted CyaA was alone sufficient for activation of mucin production through a CREB-dependent mechanism in *B. pertussis*-infected airway epithelia in vivo.

## 1. Introduction

Infections by the Gram-negative bacterium *Bordetella pertussis* cause the highly contagious respiratory illness called pertussis, or whooping cough, which used to be the primary cause of infant mortality prior to global introduction of whole cell-based pertussis vaccines seven decades ago [1]. Due to the switch to less reactogenic and less efficient acellular pertussis (aP) vaccines some two decades ago, *B. pertussis* circulation and whooping cough disease have recently resurged in most developed countries and undiagnosed mild disease and asymptomatic *B. pertussis* infections are now common in highly aP-vaccinated populations [2,3,4]. Pertussis remains the least controlled vaccine-preventable infectious disease with an estimated worldwide annual occurrence of over 24 million whooping cough cases and 160,000 pertussis-related deaths of children younger than five years of age [5]. Indeed, the *B. pertussis* bacterium is particularly well armed for immune escape and produces the potently immunosuppressive adenylate cyclase toxin (CyaA) and the more notoriously known pertussis toxin (PT), which both play a major role in hijacking of host immune response [6].

CyaA belongs to the family of RTX (Repeats in ToXin) proteins and plays a pivotal role in the initial phases of *B. pertussis* infection [7,8,9,10]. The 1706 residue-long CyaA polypeptide comprises an N-terminal cell-invasive adenylate cyclase (AC) enzyme domain of 384 residues that is linked to a 1322 residue-long pore-forming RTX hemolysin/cytolysin (Hly) moiety [11,12]. The Hly moiety binds with high affinity the CD11b subunit of the CD11b/CD18 integrin that serves as complement receptor 3 (CR3, α_M_β_2_, or Mac-1) of myeloid phagocytic cells [13,14,15,16,17]. Upon CD11b/CD18 binding, the Hly moiety inserts into the cellular membrane and mediates rapid translocation of the AC enzyme domain into cell cytosol directly across the plasma membrane of cells without the need for receptor-mediated endocytosis [18,19]. Inside cells, the AC enzyme is activated by cytosolic calmodulin and catalyzes unregulated conversion of cellular ATP to cAMP, a key second messenger [20]. This enables CyaA to instantaneously ablate the oxidative burst and complement-mediated opsonophagocytic bactericidal activities of sentinel phagocytes even at lower than 50 pM toxin concentrations [12,15,21,22,23,24,25]. The cAMP-elevating activity of CyaA thus enables bacteria to escape the surveillance of innate immunity effectors on the mucosa. Moreover, CyaA also blocks differentiation of incoming inflammatory monocytes into bactericidal macrophages and promotes de-differentiation or apoptosis of airway-resident macrophages [26,27,28,29]. Furthermore, a promiscuous low affinity binding of the toxin to glycans of glycoproteins and gangliosides on the surface of mammalian cells [14,16,30] enables CyaA to detectably elevate cAMP concentrations also in cells that do not express the CD11b/CD18 receptor of CyaA, such as the airway epithelial cells [31,32,33]. Given the rather high CyaA concentrations (>100 ng/mL) found in nasal fluids of diseased infants [33], CyaA-produced supraphysiological concentrations of cAMP most likely perturb also the innate defense mechanisms of *B. pertussis*-infected airway epithelia.

Similarly, PT promiscuously binds glycoconjugate and sialoglycoprotein receptors of various host cells and dysregulates G protein coupled receptor (GPCR)-activated cellular signaling pathways [34,35]. PT is a complex AB_5_ oligomeric toxin, with the A moiety (S1 subunit) possessing an ADP-ribosyl transferase enzyme activity and a pentameric B moiety (S2, S3, S4 and S5 subunits at 1:1:2:1 stoichiometry) that mediates cell binding. PT enters cells through receptor-mediated endocytosis within clathrin-coated pits and follows the retrograde transport pathway through the Golgi apparatus into the endoplasmic reticulum (ER) [36,37,38]. From there, the unfolded S1 subunit translocates into cell cytosol through the ER-associated protein degradation (ERAD) retrotranslocon [39,40] with the assistance of the cytosolic chaperone cyclophilin [41,42]. Once in the cytosol, the S1 subunit catalyzes the transfer of the adenosine diphosphate (ADP)-ribose moiety of nicotinamide adenine dinucleotide (NAD^+^) onto the Gα_i/o_ inhibitory subunits of the heterotrimeric G-proteins, thus locking the various isoforms of Gα_i/o_ inhibitory subunits in the inactive GDP-loaded state [43]. As a result, GPCR signaling pathways are dysregulated. Among other effects, the ADP-ribosylated Gα_i/o_ subunit isoforms cannot inhibit the activated endogenous transmembrane AC, which leads to increase of intracellular concentrations of cAMP and hijacking of protein kinase A-controlled cellular signaling pathways. The pleiotropic PT action then accounts for the key pathophysiological manifestations of pertussis [44,45], which primarily result from inhibition of leukocyte extravasation, naïve leukocyte proliferation and egress from bone marrow and lymphoid follicles into circulation (reviewed in [46]). The thus resulting pertussis-associated hyperleukocytosis then can cause fatal pulmonary hypertension and heart failure in infants due to formation of mixed lymphocyte aggregates in lung arterioles and PT intoxication of cardiac myocytes [46,47,48].

The mucus layer plays an important role in protecting airway epithelial cells from damage [49]. Mucins are heavily glycosylated proteins, where the membrane-bound mucins act predominantly as receptors, activating downstream signaling pathways leading to immune responses [50]. The gel forming mucin 5AC (Muc5AC) and mucin 5B (Muc5B) represent the major mucin species secreted by airway epithelia and account for the rheological characteristics of the mucosal layer and for the ability of the mucociliary escalator to remove the contaminated mucus from the airway [51,52]. While Muc5AC is constitutively and abundantly produced throughout the airways and is predominantly secreted by goblet cells, Muc5B is produced at lower levels and is mainly secreted by submucosal glands [53,54,55].

*B. pertussis* infection was reported to induce mucin gene transcription in bronchial epithelial cells [56] and we recently found that CyaA-produced cAMP signaling induces Muc5AC production in human bronchial epithelial cells cultured at the air–liquid interface [57]. Here, we show that the CyaA and PT toxins upregulate Muc5AC and Muc5B production in airway epithelial cell layers in vitro through a cAMP-activated PKA/CREB-dependent pathway and that CyaA alone upregulates mucus secretion in *B. pertussis*-infected mouse trachea in vivo.

## 2. Results

### 2.1. Both CyaA and PT Trigger Mucin Production by Pseudostratified Airway Epithelial Cells

**Figure 1:**
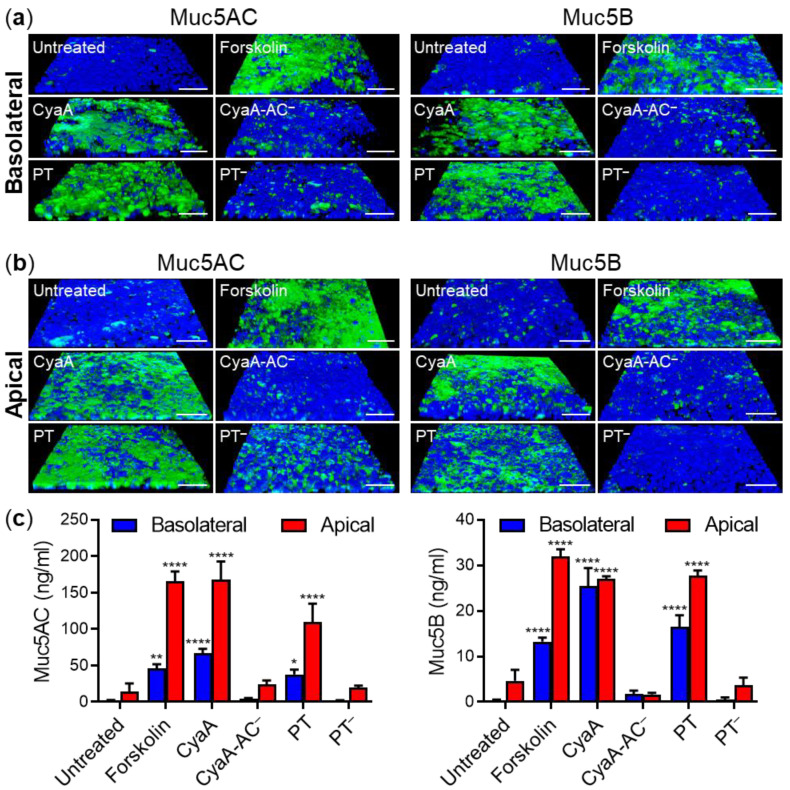
Purified CyaA and PT stimulate production of Muc5AC and Muc5B by polarized ALI-grown bronchial epithelial cells. Bronchial VA10 cell layers were treated from the basolateral (**a**) or the apical (**b**) side with 100 ng/mL of CyaA, CyaA-AC^–^, PT, PT^–^ or 20 μg/mL of forskolin for 24 h at 37 °C. The layers were fixed with 4% PFA, immunofluorescently labeled for Muc5AC and Muc5B (green) and analyzed by confocal microscopy. Cell nuclei were stained with DAPI (blue). Images of Muc5AC and Muc5B staining representative of at least three independent experiments are shown. Scale bars correspond to 50 μm on x-axis at the front edge. (**c**) The amounts of Muc5AC and Muc5B secreted onto the apical cell layers were quantified by ELISA in washes of cell layers. Bars represent means with SEM of detected Muc5AC and Muc5B amounts from at least three independent experiments. *, *p* < 0.05; **, *p* < 0.01; ****, *p* < 0.0001 compared with untreated cell layers.

Recently, we showed that an elevated cellular cAMP concentration resulting from CyaA toxin action triggers Muc5AC production in air-liquid interface (ALI)-grown differentiated VA10 human bronchial epithelial cells [57]. Since Gα_i/o_ inactivation by PT can also yield increased cAMP levels, we investigated if PT also triggers mucin production in airway epithelial cells. ALI-grown VA10 cell layers were exposed for 24 h to 100 ng/mL of PT and the cell layers were fixed for Muc5AC and Muc5B production assessment by confocal fluorescence microscopy. In parallel, the amounts of Muc5AC and Muc5B secreted onto the apical surface of cells were quantified in washes of cell layers by ELISA. For comparison, the cells were exposed to 20 µg/mL of forskolin, to activate the endogenous AC enzyme [58], or to the ATP to cAMP converting CyaA toxin (100 ng/mL). As shown in Figure 1, irrespective of whether the purified PT and CyaA toxins were applied from the basolateral or the apical side of the polarized VA10 cell layer, the action of either toxin significantly upregulated Muc5AC and Muc5B production by the epithelial cell layer to a similar extent as induced by forskolin. In contrast, cells treated with buffer or the enzymatically inactive CyaA-AC^–^ or PT-R9K/E129A (PT^–^) toxoids produced only basal levels of Muc5AC and Muc5B. Hence, induction of mucin production in differentiated VA10 bronchial epithelial cells was strictly due to the catalytic activities of the toxins (cAMP-generating adenylyl cyclase activity of CyaA and ADP-ribosylating activity of the S1 subunit of PT), which directly (CyaA) or indirectly (PT) increase cellular cAMP levels.

### 2.2. CyaA and PT Trigger Phosphorylation of CREB in VA10 Cell Layers

**Figure 2:**
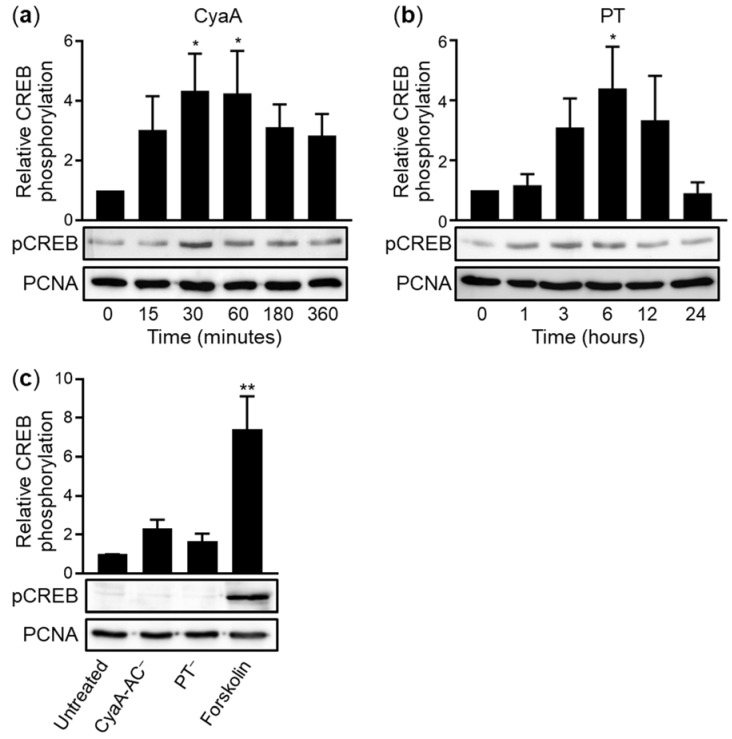
Purified CyaA and PT stimulate phosphorylation of CREB. Either 100 ng/mL of CyaA (**a**) or 100 ng/mL of PT (**b**) were added basolaterally to ALI-grown polarized VA10 cell layers for the indicated times. For comparison, 100 ng/mL of CyaA-AC^–^ for 30 min, or 100 ng/mL of PT^–^ for 6 h, were added as negative controls and 20 μg/mL of forskolin for 30 min was added as a positive control, respectively (**c**). Cell layers were lysed and nuclear lysates were analyzed by Western blotting. Activating phosphorylation of the Ser133 residue of CREB (pCREB) was detected using a pCREB-specific antibody. Immunoblots developed with anti-PCNA (proliferating cell nuclear antigen) antibody served as loading controls. Data represent mean with SEM from eight (**a**) or three (**b**,**c**) independent experiments. * *p* < 0.05; ** *p* < 0.01 compared with untreated cell layers (time 0 in (**a**,**b**)).

The cAMP response element binding protein (CREB) was previously found to regulate expression of the *Muc5AC* and *Muc5B* genes in airway epithelial cells [59]. This ubiquitous transcription factor stimulates transcription following phosphorylation of its serine 133 (Ser133) residue by protein kinase A (PKA) [60]. Since PKA itself is activated by cAMP produced directly by CyaA, or indirectly elevated by PT action [61,62], we examined if upregulation of mucin production involved the PKA-CREB signaling axis. VA10 cell layers were exposed to 100 ng/mL of CyaA or PT from the basolateral side and phosphorylation of CREB over time of toxin action was assessed by immunoblots of nuclear extracts of cells with an anti-phospho-CREB (Ser133) antibody. Indeed, CyaA triggered a fourfold increase of phosphorylated CREB (pCREB) levels already within 30 min of action and increased pCREB levels persisted in CyaA-exposed cells for at least 6 h (Figure 2a). In contrast, PT triggered a slower increase of pCREB level that peaked in 6 h from PT addition and decreased to the basal level within 24 h (Figure 2b). As expected, the CyaA-AC^–^ and PT^–^ toxoids did not elicit any CREB phosphorylation (Figure 2c).

**Figure 3:**
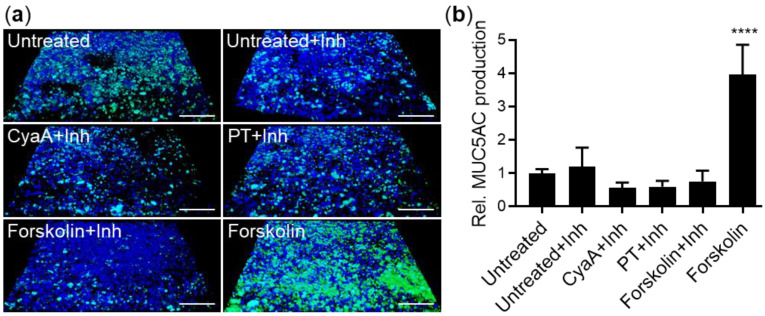
CyaA- and PT-mediated increase in mucin production is blocked by CREB inhibitor. (**a**) ALI-grown VA10 epithelial cell layers were pretreated from the basolateral side for 1 h with 10 μM 666-15-Calbiochem inhibitor (Inh) of CREB-mediated gene transcription and then incubated with 100 ng/mL of CyaA, 100 ng/mL of PT or 20 μg/mL of forskolin for 24 h at 37 °C. The layers were fixed with 4% PFA, stained for Muc5AC (green) and analyzed by confocal microscopy. Nuclei were stained with DAPI (blue) and the VA10 cell layer treated with 20 μg/mL of forskolin without the inhibitor is shown as a positive control. Scale bars correspond to 100 μm on x-axis at the front edge. (**b**) Muc5AC staining was quantified on representative images using the software ImageJ. Bars represent means with SEM of relative Muc5AC production from at least three independent experiments. **** *p* < 0.0001 compared with untreated cell layers.

Moreover, no upregulation of Muc5AC production was observed (Figure 3) when the ALI-grown epithelial cell layers were preincubated for 1 h prior to toxin addition with the 666-15-Calbiochem inhibitor of CREB-mediated gene transcription [63,64]. Hence, it can be concluded that the PT and CyaA toxins induced mucin production in polarized human bronchial epithelial cells by activation of PKA-mediated phosphorylation of CREB.

### 2.3. Only Secretion of CyaA Triggers Mucin Production in B. pertussis-Infected VA10 Cell Layers and in Infected Mouse Tracheas In Vivo

**Figure 4:**
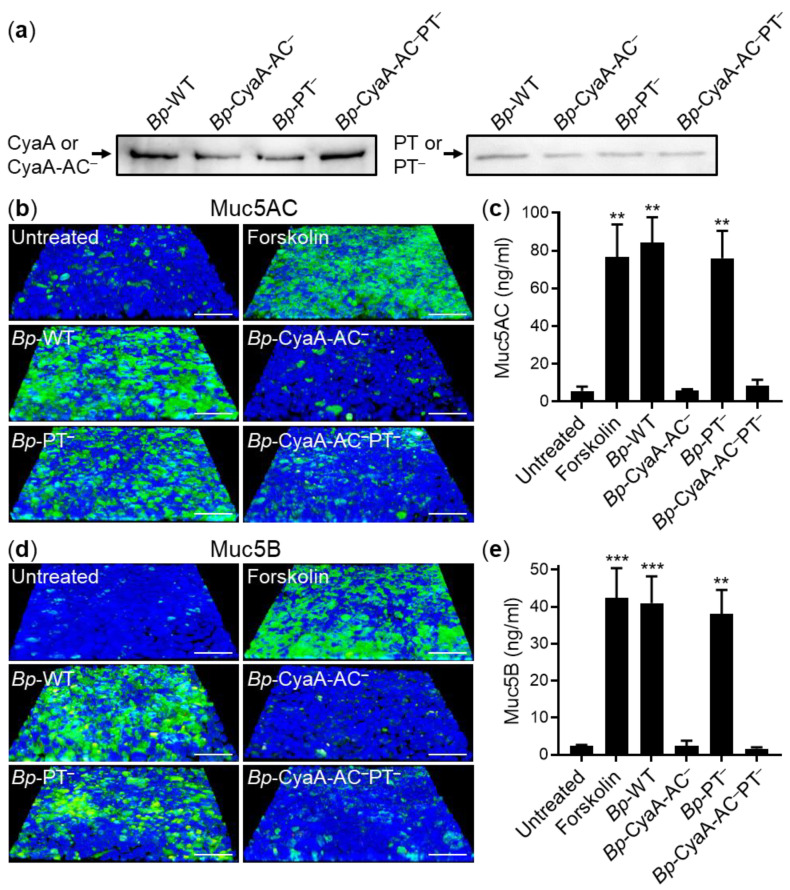
*B. pertussis*-secreted CyaA alone triggers Muc5AC and Muc5B production by infected epithelial cells. (**a**) *B. pertussis* Tohama I (*Bp*-WT) and its isogenic mutant strains (*Bp*-CyaA-AC^–^, *Bp*-PT^–^ and *Bp*-CyaA-AC^–^PT^–^) secrete the toxins CyaA and PT and/or their respective toxoids. Bacteria were cultured in complete SS medium in the presence of 2 mM CaCl_2_ to an OD_600_ of 1 and the secreted proteins were detected by Western blotting. Monoclonal antibodies specific for CyaA and CyaA-AC^–^ or for the S1 subunit of PT and PT^–^ were used to detect the secreted proteins. (**b**–**e**) ALI-grown polarized VA10 cell layers were infected from the apical side with *Bp*-WT or its isogenic mutant strains at an MOI of 50:1 for 24 h at 37 °C. The layers were fixed with 4% PFA, immunofluorescently labeled for Muc5AC (**b**) and Muc5B (**d**) (green) and analyzed by confocal microscopy. Cell nuclei were stained with DAPI (blue) and the VA10 cell layers apically treated with 20 μg/mL of forskolin are shown as positive controls. Representative images of Muc5AC and Muc5B staining from at least five independent experiments are shown. Scale bars correspond to 50 μm on x-axis at the front edge. The amounts of Muc5AC (**c**) and Muc5B (**e**) secreted onto the apical cell layers were quantified by ELISA. Bars represent means with SEM of Muc5AC and Muc5B production from three independent experiments. ** *p* < 0.01; *** *p* < 0.001 compared with untreated cell layers.

We next examined if the CyaA and PT amounts secreted during *B. pertussis* infections of airway epithelial cell layers are sufficient for triggering mucin production. Therefore, VA10 cell layers were infected at a multiplicity of infection (MOI) of 50:1 from the apical side with wild-type *B. pertussis* Tohama I strain (*Bp*-WT) that produces both CyaA and PT toxins, or by its *Bp*-CyaA-AC^–^, *Bp*-PT^–^ or *Bp*-CyaA-AC^–^PT^–^ toxoid-secreting isogenic mutants, respectively (Figure 4a). After 24 h, the cell layers were fixed and stained for Muc5AC or Muc5B (Figure 4b,d) and in parallel the amounts of apically secreted Muc5AC and Muc5B were determined by ELISA (Figure 4c,e). As shown in Figure 4b–e, infection by *Bp*-WT, or the *Bp*-PT^–^ mutant producing intact CyaA, induced similar Muc5AC and Muc5B production in polarized VA10 cell layers as the treatment with forskolin. In contrast, epithelial cell infections with the *Bp*-CyaA-AC^–^ mutant that secreted the CyaA-AC^–^ toxoid but produced active PT, failed to trigger any increase of Muc5AC and Muc5B production above the level of mucin production by uninfected cells (Figure 4b–e). It can thus be concluded that the CyaA toxin amounts secreted by *B. pertussis* were alone sufficient for induction of mucin production in infected VA10 cell layers and the secreted PT on its own did not trigger any mucin production under the used experimental conditions in vitro.

To corroborate this observation under conditions of *B. pertussis* infection in vivo, BALB/cByJ mice were infected intranasally with ~8 × 10^5^ colony forming units (CFU) of *Bp*-WT, or of the toxoid-producing isogenic mutant strains. Bacterial colonization on days 5 and 14 of infection was determined by plating homogenates of aseptically removed tracheas on BG agar and in parallel the production of mucus in the infected tracheas was visualized on formalin-fixed histological sections by anti-Muc5AC immunohistochemical detection of Muc5AC production and by Alcian blue staining for acidic mucopolysaccharides and sialomucins, respectively.

**Figure 5:**
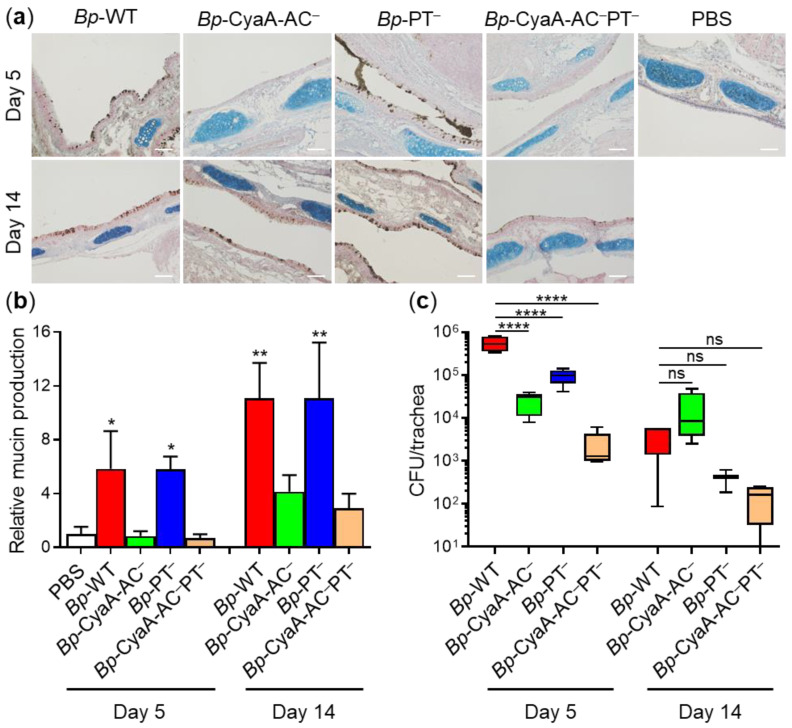
*B. pertussis*-secreted CyaA alone triggers mucin production in infected mouse tracheas in vivo. Specific pathogen-free BALB/cByJ mice were intranasally infected with 50 µL of suspensions containing ~8 × 10^5^ CFU/mouse of the parental strain *Bp*-WT, or of the isogenic mutant strains *Bp*-CyaA-AC^–^, *Bp*-PT^–^ and *Bp*-CyaA-AC^–^PT^–^, respectively. Animals were sacrificed on day 5 or 14 by cervical dislocation. Tracheas were aseptically removed, fixed with 4% PFA and upon embedding and antigen retrieval, the immunohistochemical staining of histological sections for Muc5AC was performed followed by Alcian blue staining of acidic mucopolysaccharides and sialomucins. (**a**) Tracheas with stained mucins were visualized by light microscopy and representative images are shown. Scale bars, 100 μm. (**b**) Tracheas were scanned using an automated slide scanner and the amounts of tracheal mucins were quantified using the QuPath software. Bars represent means with SEM of relative mucin amounts from two independent experiments (4–5 tracheas in each group on day 5; 2–5 tracheas on day 14). The mean amount of mucin in tracheas of uninfected animals (PBS) on day 5 was taken as 1. * *p* < 0.05; ** *p* < 0.01 compared with PBS-treated animals. (**c**) Tracheal colonization was quantified by plating serially diluted homogenates of aseptically extracted tracheas (5 tracheas in each group on day 5; 4 tracheas on day 14) on Bordet-Gengou agar plates and *B. pertussis* CFUs were counted after 5 days of colony growth at 37 °C. **** *p* < 0.0001; ns, non-significant.

As documented in Figure 5a and quantified in Figure 5b, infection by the *Bp*-WT strain significantly increased the amount of mucin accumulating in the tracheas of animals on day 5 of infection, when the CFU count of *Bp*-WT in the tracheas reached ~5.6 × 10^5^ CFU/trachea (Figure 5c). A further increase in the amount of mucin accumulated in the tracheas of *Bp*-WT-infected animals was observed on day 14, despite the fact that the CFU counts of the *Bp*-WT bacteria already decreased to ~4.2 × 10^3^ CFU/trachea. As also shown in Figure 5a,b, the *Bp*-PT^–^ mutant secreting the active CyaA toxin increased the amount of mucin accumulated in the tracheas to a similar extent as the infection by the parental *Bp*-WT strain, despite a reduced trachea colonization capacity (~9.6 × 10^4^ vs. ~5.6 × 10^5^ CFU on day 5 and ~4.1 × 10^2^ vs. ~4.2 × 10^3^ CFU on day 14; Figure 5c). In contrast, the *Bp*-CyaA-AC^–^ mutant, secreting active PT and reaching insignificantly higher CFU counts in mouse tracheas on day 14 than *Bp*-WT, failed to increase mucin levels in infected tracheas on day 5 at all and provoked only insignificant increase in mucin production in the tracheas by day 14. Similarly, infection by the double *Bp*-CyaA-AC^–^PT^–^ mutant, which colonized the mouse trachea only poorly, did not provoke any observable upregulation of tracheal mucin production over the level of uninfected control mice. Collectively, these data show that the production of CyaA alone accounted for increased mucin production in the tracheas of *B. pertussis*-infected mice and PT activity contributed only little, if at all, to the induction of mucin secretion in the airways of infected animals.

## 3. Discussion

We show that by activating PKA-specific phosphorylation of the transcription factor CREB, the CyaA-generated cAMP upregulates secretion of mucins in *B. pertussis*-infected airway epithelia.

The cAMP/PKA-activated transcription factor CREB was previously shown to play a critical role in regulation of expression of the *Muc5AC* and *Muc5B* genes in airway epithelial cells [59]. We thus first examined whether CyaA and PT action on ALI-grown polarized VA10 cells upregulates production of Muc5AC and Muc5B. While CyaA delivers into cells an AC enzyme that directly converts ATP to cAMP, PT elevates cellular cAMP concentrations only indirectly by relieving the regulation of the endogenous transmembrane AC enzyme of cells. As shown here for the first time, addition of 100 ng/mL of purified PT triggered a significant increase of CREB phosphorylation in polarized VA10 airway epithelial cells within 6 h (see Figure 2). In contrast, exposure to CyaA toxin triggered the maximal increase in the levels of phosphorylated CREB already within the first 30 min. This difference in kinetics of CyaA and PT-mediated activation of CREB can be readily explained by the different kinetics and modes of cell entry and action of the two toxins. Indeed, CyaA translocates its enzymatic AC domain directly across the plasma membrane of cells with a very short half-time of only about ~30 s [65]. As a result, the extremely active AC enzyme of CyaA (catalytic number of ~2000 s^−1^) starts converting cytosolic ATP to cAMP in the submembrane space of cells near-instantly upon toxin binding to cells. In contrast, the cAMP-elevating action of PT involves several time-consuming steps involved in endocytosis, retrograde transport, translocation of the enzymatic subunit across the ER membrane into cell cytosol and finally the inactivation of the Gα_i/o_ subunits by PT-catalyzed ADP-ribosylation. Only then the inactivation of the Gα_i/o_ can occur and eventually relieve the inhibition of the endogenous AC, thus eliciting an increase of cAMP levels in PT-treated cells with an important delay [66,67].

Moreover, in vitro infection experiments with *B. pertussis* mutants on polarized epithelial cell layers, as well as in vivo infections of mouse tracheas, revealed that only the *B. pertussis* strains secreting an active CyaA toxin were able to upregulate mucin production in airway epithelia. The *Bp*-CyaA-AC^–^ mutant, producing active PT and an enzymatically inactive CyaA-AC^–^ toxoid, failed to upregulate mucin secretion by airway epithelial cells. It thus appears that the amounts of PT produced in vivo are insufficient for contributing any noticeably to the CyaA/cAMP-dependent upregulation of mucin production through the PKA/CREB axis in the course of airway infections.

Previously, we and others have observed that CyaA added to ALI-grown airway epithelial cell layers increased total cellular cAMP concentrations more efficiently when applied from the basolateral side than from the apical side [57,68]. However, in the present work we found that physiologically relevant (100 ng/mL) CyaA amounts [33] triggered about ~2.5 times higher Muc5AC secretion when the toxin was added from the apical side of the epithelial layer (see Figure 1). This suggests that formation of CyaA-produced cAMP gradients in the apical submembrane compartment yields more efficient activation of CREB than CyaA-catalyzed cAMP formation in the basolateral submembrane compartment. 

Additional work is currently underway to explore in more detail the role played by CyaA-mediated upregulation of mucin secretion in the capacity of *B. pertussis* to colonize the upper airways and proliferate to sufficient density to maximize the probability of transmission to new hosts within mucus-containing aerosol droplets. While the mucus layer contains antimicrobial peptides and proteins restricting bacterial growth, a CyaA-upregulated increase in its thickness would alter its viscosity and elasticity and thereby compromise the capacity of the mucociliary escalator to remove the *B. pertussis*-infected mucus layer from the airways. It is plausible to speculate that this may support bacterial proliferation to concentrations enabling transmission to new hosts by droplet shedding, as previously hypothesized [56,69].

## 4. Materials and Methods

### 4.1. VA10 Cell Layers

The E6/E7 viral oncogene immortalized human bronchial epithelial cell line VA10 was obtained as a kind gift from Dr. Gudmundur H. Goodmundsson (Biomedical Centre, University of Iceland, Reykjavik, Iceland) and was used between passage 18 and 25. The cells were cultured in LHC9 growth medium (Thermo Fisher Scientific, Waltham, MA, USA) in 75 cm^2^ flasks in a humidified, 5% CO_2_–95% atmospheric air incubator at 37 °C. Medium was changed twice a week and the cells were passaged weekly at 1:3 split ratio by using 10 mL of EDTA-trypsin solution (1:1). After reaching confluency, the cells were seeded on Transwell permeable supports (0.4 μm pore size, polyester membrane; Corning Costar Corporation, Cambridge, MA, USA) with a seeding density of 200,000/cm^2^. The cells were kept in Dulbecco’s modified Eagle’s medium (DMEM; Sigma-Aldrich, St. Louis, MO, USA) mixed with Ham’s Nutrient Mixture F12 medium (F12; Sigma-Aldrich, St. Louis, MO, USA) in a ratio of 1:1 (*v*/*v*) and supplemented with 2% Ultroser G (PALL Life Sciences, New York, NY, USA) and antibiotic-antimycotic solution (Sigma-Aldrich, St. Louis, MO, USA) for 4–7 days with medium both on the basolateral side and on the apical side. Then, the medium was removed from the apical side and replaced only on the basolateral side every two days with medium used for ALI cultures containing DMEM/F12 (1:1), 1% (*w*/*v*) gentamycin (Thermo Fisher Scientific, Waltham, MA, USA) and UltroserG serum substitute (PALL Life Sciences, New York, NY, USA). The cells in ALI culture were incubated in a humidified, 5% CO_2_–95% atmospheric air incubator for ~14 to 21 days at 37 °C. Trans-epithelial electrical resistance (TEER) was measured in all mature VA10 cell layers using a Millicell-ERS volt-ohm meter (Merck Life Science, Darmstadt, Germany) and only the layers that generated TEER of at least 350 Ω cm^2^ (Transwell inserts with a diameter of 12 mm) or of at least 1100 Ω cm^2^ (Transwell inserts with a diameter of 6.5 mm) were used for experiments.

### 4.2. Intoxication Experiments

The cell layers of VA10 cells cultured in ALI were washed 2-times with phosphate-buffered saline (PBS) for 30 min to remove baseline secreted mucus [70] and then left to rest for 1 h prior to experiments. Next, the cell layers were apically or basolaterally treated with 100 ng/mL of LPS-free purified CyaA or catalytically inactive toxoid CyaA-AC^–^ (both produced and purified as previously described [71]), or with 100 ng/ml of purified PT (a kind gift of Dr. Umesh Shaligram from Serum Institute of India Pvt. Ltd., Pune, India) or catalytically inactive PT^–^ (List Biological Laboratories, Campbell, CA, USA), or with 20 µg/mL of forskolin (positive control; Sigma-Aldrich, St. Louis, MO, USA). In some experiments, the cell layers were pre-treated for 1 h before addition of toxins or toxoids with 10 μM 666-15-Calbiochem (Merck Life Science, Darmstadt, Germany), a specific inhibitor of CREB-mediated gene transcription [63]. After incubation at different times in a humidified, 5% CO_2_–95% atmospheric air incubator at 37 °C, the cells layers were processed to be analyzed by confocal microscopy, ELISA, or Western blot.

### 4.3. Confocal Microscopy

The treated VA10 cell layers on the Transwell permeable supports were fixed with PBS-buffered (pH 7.4) formaldehyde (4% PFA) solution and permeabilized with 0.1% Triton X-100 in PBS for 5 min at room temperature. After permeabilization, the membranes were blocked with 5% bovine serum albumin (BSA) in PBS for 1 h at room temperature followed by incubation overnight with primary monoclonal mouse anti-Muc5AC and Muc5B antibodies (clones 45M1 and 5B19-2E, respectively; Thermo Fisher Scientific, Waltham, MA, USA) diluted 1:200 in PBS with 2% BSA. The membranes were then washed three times with PBS and incubated with Alexa Fluor 488-conjugated secondary anti-mouse IgG antibody (Thermo Fisher Scientific, Waltham, MA, USA) diluted 1:200 in PBS with 2% BSA along with DAPI (2 µg/mL) for 1 h. Finally, the membranes were washed with PBS, rinsed once with distilled water and mounted on clean microscopic slides in Vectashield mounting medium (Vector Laboratories, Burlingame, CA, USA). Immunofluorescence images were obtained using Olympus FV-1000 confocal microscope (Olympus Corporation, Tokyo, Japan) and processed using the software ImageJ (version 1.51, U. S. National Institutes of Health, Bethesda, MD, USA).

### 4.4. Muc5AC and Muc5B ELISA

Secreted mucus was collected by washing the apical side of the treated VA10 cell layers twice with 250 µL of PBS and the Muc5AC and Muc5B mucin amounts were quantified using Muc5AC and Muc5B-specific ELISA kits according to manufacturer’s instructions (Novus Biologicals, LLC- Bio-Techne Ltd., Abingdon, UK).

### 4.5. Immunodetection of Phosphorylated CREB

The treated VA10 cell layers were washed once with chilled PBS and incubated with 500 µL of hypotonic buffer (20 mM Tris-HCl (pH 7.4), 10 mM NaCl, 3 mM MgCl_2_) for 15 min at 4 °C. The cell suspensions were then scraped off the membrane inserts, collected in Eppendorf tubes and centrifuged (10 min, 1000 *g*) to separate the cytosolic fraction from the nuclei. The pellets with nuclei were re-suspended with 90 µL of Triton X lysis buffer (50 mM Tris-HCl (pH 7.4), 150 mM NaCl, 1% Triton X-100, 1 mM Na_3_VO_4_, 10 mM NaF, 1x Complete Mini Protease Inhibitor Cocktail (Roche, Mannheim, Germany)) and incubated for 30 min at 4 °C. The lysates were cleared by centrifugation (30 min, 20,000 *g*) and the supernatants containing the nuclear protein fractions were collected, separated by SDS-polyacrylamide gel electrophoresis (SDS-PAGE) on an 8.5% gel and transferred onto a PVDF membrane (Merck Life Science, Darmstadt, Germany). The membrane was blocked with 5% BSA in PBS and probed with an antibody against pCREB (clone 1B6; Cell Signaling Technology, Danvers, MA, USA) diluted 1:500 in PBS with 2% BSA, or with an antibody against proliferating cell nuclear antigen (PCNA; clone PC10; EXBIO, Vestec, Czech Republic) diluted 1:1000 in PBS with 2% BSA overnight, respectively. After washing, the detected proteins were decorated by anti-mouse IgG-peroxidase conjugate (Sigma-Aldrich, St. Louis, MO, USA) diluted 1:3000 in PBS with 2% BSA for 1 h and visualized by chemiluminescence signal using SuperSignal West Femto Maximum Sensitivity Substrate (Thermo Fisher Scientific, Waltham, MA, USA), using a G:BOX Chemi XRQ gel doc system instrument (Syngene, Cambridge, UK). The chemiluminescence signal was quantified using ImageJ processing and analysis software (version 1.51, U. S. National Institutes of Health, Bethesda, MD, USA).

### 4.6. B. pertussis Strains

The wild type *B. pertussis* Tohama I strain (CIP 81.32) was obtained from the Collection of Institute Pasteur (Paris, France). The CyaA-AC^–^ and PT-R9K/E129G (PT^–^) toxoid-secreting mutants were constructed using the allelic exchange plasmid pSS4245 and were previously described [10,28]. The *B. pertussis* mutant strains *Bp*-CyaA-AC^–^ and *Bp*-CyaA-AC^–^PT^–^ contain an insertion of a Gly-Ser dipeptide between amino acid residues 188 and 189 of CyaA, resulting in production of an enzymatically inactive toxoid (CyaA-AC^–^) unable to convert ATP to cAMP [10,72]. The *B. pertussis* mutant strains *Bp*-PT^–^ and *Bp*-CyaA-AC^–^, PT^–^ contain two point substitutions R9K and E129G in the enzymatic subunit S1 of PT, resulting in production of an enzymatically inactive toxoid (PT^–^) [28,73]. All *B. pertussis* strains were grown on Bordet-Gengou agar (BGA) plates (Becton Dickinson, Prague, Czech Republic) containing 15% defibrinated sheep blood (LabMediaServis, Jaromer, Czech Republic) for 3–7 days at 37 °C in a 5% CO_2_–95% atmospheric air incubator. Colonies from a fresh plate were re-suspended to an OD_600_ of 0.2 in modified Stainer-Scholte (SS) medium supplemented with 5 g/L of casamino acids (Difco, BD, Franklin Lakes, NJ, USA) and 1 g/L of 2-hydroxypropyl-β-cyclodextrin (complete SS medium). The bacteria were grown overnight at 37 °C with shaking to an OD_600_ of 1 (~2 × 10^9^ CFU/mL).

### 4.7. Immunodetection of the CyaA and PT Toxins and Toxoids

To confirm secretion of the CyaA and PT toxins and their respective toxoids by the used *B. pertussis* strains, bacteria were grown at 37 °C in 3 mL of complete SS medium supplemented with 2 mM CaCl_2_ to facilitate secretion of CyaA or CyaA-AC^–^ [74]. Bacterial suspensions in the exponential growth phase (OD_600_ = 1) were centrifuged at 13,000 *g* for 5 min. The supernatant was collected and the secreted proteins were denatured with Laemmli loading buffer and separated by SDS-PAGE (7.5% gel for CyaA and 12.5% gel for PT). The separated proteins were transferred to a nitrocellulose membrane (Immobilon-NC, GE Healthcare, Chicago, IL, USA) that was subsequently blocked with 5% nonfat milk in TNT buffer (50 mM Tris-HCl (pH 8.0), 150 mM NaCl, 0.05% Tween-20). The membranes were incubated with a monoclonal antibody specific for CyaA (clone 9D4; diluted 1:3000) [75] overnight at 4 °C or with a monoclonal antibody specific for S1 subunit of PT (Santa Cruz Biotechnology, Dallas, TX, USA; diluted 1:500) diluted in 3% nonfat milk in TNT buffer. The chemiluminescence signal was developed using a SuperSignal West Femto substrate (Thermo Fisher Scientific, Waltham, MA, USA) after a 1-h incubation of the membrane with a horseradish peroxidase-conjugated goat anti-mouse secondary antibody (GE Healthcare, Chicago, IL, USA) and scanned by a G:BOX Chemi XRQ gel doc system instrument (Syngene, Cambridge, UK).

### 4.8. Bacterial Infection of VA10 Cell Layers

Bacterial suspensions were diluted in DMEM/F12 with 2% UltroSerG (PALL Life Sciences, New York, NY, USA) and no antibiotics to ~2 × 10^7^ CFU/mL and incubated further at 37 °C for 1 h before addition to the VA10 cell layers at an MOI of 50.

### 4.9. Mice Infections and Ethics Statement

Specific pathogen-free BALB/cByJ mice were purchased from Charles River Laboratories (Ecully, France) and housed at the animal facility of the Institute of Molecular Genetics of the CAS, v. v. i., Prague, Czech Republic. Handling of animals was performed according to the Guidelines for the Care and Use of Laboratory Animals, the Act of the Czech National Assembly, Collection of Laws no. 246/1992. Animal experiments were approved by the Animal Welfare Committee of the Institute of Molecular Genetics of the CAS, v. v. i. (protocol code: 10/2020, date of approval: 19 February 2020).

Female, 5 weeks of age BALB/cByJ mice were anesthetized by intraperitoneal (i.p.) injection of ketamine (80 mg/kg) and xylazine (8 mg/kg) and inoculated intranasally with 50 μL of *B. pertussis* cell suspensions grown overnight in modified SS liquid medium and diluted in PBS. The bacterial suspensions contained on average 8 × 10^5^ viable CFU in 50 μL, as assessed by serial dilutions and plating on BG agar plates. To determine the bacterial load from tracheas, mice were sacrificed by cervical dislocation 5 and 14 days after infection. Whole tracheas were aseptically removed and homogenized in PBS with tissue grinders. The homogenates were plated directly or serially diluted on BG agar plates and CFUs were counted after 5 days of growth at 37 °C in a 5% CO_2_–95% atmospheric air incubator.

### 4.10. Staining and Quantification of Tracheal Mucins

Tracheas for histopathology were collected at days 5 and 14 after infection with *B. pertussis* strains and fixed in 4% PFA solution that was exchanged for 70% (*v*/*v*) denatured ethanol after 24 h. Tracheas were processed with an ASP 6025 machine and embedded in paraffin with an EG 1150 C machine (both Leica Biosystems, Nussloch, Germany). The blocks were subsequently cut at a 2.5-μm thickness using the LEICA RM 2255 Microtome (Leica Biosystems, Nussloch, Germany). The sections were placed onto positively charged microscope slides (Thermo Fisher Scientific, Waltham, MA, USA) for immunohistochemistry (IHC). The slides were dried for 1 h at 65 °C and deparaffinized using an automated LEICA ST5020 machine (Leica Biosystems, Nussloch, Germany). The tissue sections were rehydrated and antigen retrieval was performed with HIER EDTA Buffer pH 8.0 (Zytomed GmbH, Berlin, Germany) for 15 min at 110 °C. After rehydration and antigen retrieval, sections were stained with a primary monoclonal anti-Muc5AC antibody (clone 45M1; Thermo Fisher Scientific, Waltham, MA, USA) at a 1:200 dilution with overnight incubation in a humid chamber at 4 °C followed by incubation with an HRP-conjugated anti-mouse IgG (Zytomed GmbH, Berlin, Germany) for 30 min at room temperature. The detection was done using a DAB Substrate kit (Abcam, Cambridge, UK) for 2 min at room temperature. The IHC-stained slides were then stained with Alcian blue (pH 2.5) for 30 min by immersion to stain sulfated and carboxylated acid mucopolysaccharides and sulfated and carboxylated sialomucins. The slides were then counterstained with Nuclear Fast Red solution for 5–10 min by immersion and mounted with Pertex mounting medium (Leica Multistainer ST5020, Leica Biosystems, Nussloch, Germany). For the first evaluation, samples were inspected at a Carl Zeiss Scope.A1 light microscope (Carl Zeiss, Göttingen, Germany). Full-slide scans of tracheas were obtained by an AxioScan.Z1 automated slide scanner (Carl Zeiss, Göttingen, Germany) at a 20× magnification using the Zeiss ZEN Blue software (version 11.0, Carl Zeiss, Göttingen, Germany). The amounts of stained mucins on the tracheal epithelial layers were quantified in a blinded manner independently by two different pathologists using the QuPath software (version 0.2.0, Queen’s University Belfast, Belfast, Northern Ireland, UK) [76]. Quantification was performed on multiple pictures taken from full-slide scans imported into QuPath, in which each sample was linked to a single project. An image type was set as ‘brightfield H-DAB’. To perform image analysis, the epithelial layer of tracheas was manually selected in each image via the ‘brush selection tool’ of the software. The selected areas (stored as ‘annotations’ in the software) were then automatically analyzed by the ‘positive cell detection’ tool of QuPath, setting ‘optical density sum’ as detection image method (cell: DAB OD mean). Quantification was performed by assigning the same threshold to each image and the relative proportion of the detected mucins (positive area) to the total area was calculated.

### 4.11. Statistical Analysis

Results were expressed as the arithmetic mean ± standard error of the mean (SEM). One- or two-way ANOVA was used to calculate statistical significance (GraphPad Prism 9.1.1; GraphPad Software, La Jolla, CA, USA).
